# Authors’ Reply: Enhancing Team-Based Learning in Virtual Environments: The Role of Avatar Agency and Immersive Social Presence

**DOI:** 10.2196/93060

**Published:** 2026-03-30

**Authors:** Darunee Sripadungkul, Suhattaya Boonmak, Monsicha Somjit, Narin Plailaharn, Wimonrat Sriraj, Polpun Boonmak

**Affiliations:** 1 Anesthesiology Department, Faculty of Medicine, Khon Kaen University, Ni Muang, Khon Kaen, 40002, Thailand, 66 842003331

**Keywords:** anesthesia, computer-assisted instruction, distance, education, internet, learning, medical, problem-based learning, students, teaching, virtual reality

Thank you for the thoughtful comments [1] on our randomized controlled trial comparing web-based virtual environment (WBVE) versus face-to-face delivery for team-based learning (TBL) of anesthesia techniques [2]. We appreciate the emphasis on immersion, avatar agency, and social presence as potential determinants of learner experience in virtual settings.

We agree that reduced social cues are a plausible contributor to the satisfaction gap. In our study, WBVE was delivered through a desktop-based, nonimmersive environment, which inherently limits nonverbal communication . This likely affects perceived interaction quality during TBL and suggests that lower satisfaction may reflect constraints of the interface rather than a limitation of virtual pedagogy itself [2].

Our primary objective was to evaluate a delivery approach that is low-cost, scalable for large cohorts, and logistically feasible for undergraduate medical students. These implementation priorities guided our selection of a desktop-based WBVE as a practical alternative to face-to-face [2]. While head-mounted display–based immersive 3D virtual reality (VR) may increase immersion and avatar agency, it also introduces additional equipment, staffing, and cost demands that can limit scalability [3-5]. Because we did not include a head-mounted display VR arm, we cannot determine whether immersive VR would eliminate the satisfaction difference. Future studies should directly compare desktop WBVE and immersive VR while measuring social presence, usability, cognitive load, feasibility, and cost.

Learner satisfaction in our trial was assessed across four domains: (1) learning topic, (2) learning process (including instructor facilitation and delivery technique), (3) learning outcomes, and (4) overall course satisfaction. Ratings were generally high in both groups, but the largest differences clustered in learning process elements most sensitive to delivery modality on discussion flow, idea sharing, and spontaneous expression. We interpret this pattern as reduced social presence in nonimmersive WBVE [2].

Regarding avatar settings, the WBVE session was delivered on the Spatial platform using a desktop setup. On first log-in, each participant created an account and entered the virtual space as a keyboard-controlled avatar. Teams collaborated synchronously using Spatial’s voice and text chat while completing standardized TBL modules. We did not experimentally manipulate avatar agency (eg, avatar customization or embodiment). Therefore, our findings apply to this specific nonimmersive, desktop-based WBVE implementation [2].

In summary, our findings support desktop WBVE as a scalable approach with comparable knowledge gains while highlighting interaction-related limitations that should be targeted in future virtual TBL design and evaluation.

## Abbreviations

TBL: team-based learning
VR: virtual reality
WBVE: web-based virtual environment
